# Data-Independent Acquisition Mass Spectrometry Analysis of FFPE Rectal Cancer Samples Offers In-Depth Proteomics Characterization of the Response to Neoadjuvant Chemoradiotherapy

**DOI:** 10.3390/ijms242015412

**Published:** 2023-10-21

**Authors:** Aleksandra Stanojevic, Martina Samiotaki, Vasiliki Lygirou, Mladen Marinkovic, Vladimir Nikolic, Suzana Stojanovic-Rundic, Radmila Jankovic, Antonia Vlahou, George Panayotou, Remond J. A. Fijneman, Sergi Castellví-Bel, Jerome Zoidakis, Milena Cavic

**Affiliations:** 1 Department of Experimental Oncology, Institute for Oncology and Radiology of Serbia, Pasterova 14, 11000 Belgrade, Serbia; astefanovic496@gmail.com (A.S.); jankovicr@ncrc.ac.rs (R.J.); 2 Institute for Bioinnovation, Biomedical Sciences Research Center “Alexander Fleming”, Fleming 34, 166 72 Vari, Greece; samiotaki@fleming.gr (M.S.); panayotou@fleming.gr (G.P.); 3 Department of Biotechnology, Biomedical Research Foundation, Academy of Athens, 4 Soranou Ephessiou Street, 115 27 Athens, Greece; vlygirou@bioacademy.gr (V.L.); vlahoua@bioacademy.gr (A.V.); izoidakis@bioacademy.gr (J.Z.); 4 Clinic for Radiation Oncology and Diagnostics, Department of Radiation Oncology, Institute for Oncology and Radiology of Serbia, Pasterova 14, 11000 Belgrade, Serbia; mladen309@gmail.com (M.M.); stojanovics@ncrc.ac.rs (S.S.-R.); 5 Faculty of Medicine, University of Belgrade, Dr Subotica 8, 11000 Belgrade, Serbia; 6 Clinic for Medical Oncology, Institute for Oncology and Radiology of Serbia, Pasterova 14, 11000 Belgrade, Serbia; vladimirdnikolic@gmail.com; 7 Department of Pathology, The Netherlands Cancer Institute, Plesmanlaan 121, 1066 CX Amsterdam, The Netherlands; r.fijneman@nki.nl; 8 Gastroenterology Department, Fundació Clínic per la Recerca Biomèdica-Institut d’Investigacions Biomèdiques August Pi i Sunyer (FRCB-IDIBAPS), C/del Rosselló, 149, 08036 Barcelona, Spain; sbel@recerca.clinic.cat; 9 Centro de Investigación Biomédica en Red de Enfermedades Hepáticas y Digestivas (CIBERehd) Almagro, 3, 28029 Madrid, Spain; 10 Hospital Clínic, University of Barcelona, C/del Villarroel, 170, 08036 Barcelona, Spain; 11 Department of Biology, National and Kapodistrian University of Athens, Panepistimíou 30, 106 79 Athens, Greece

**Keywords:** data-independent acquisition mass spectrometry, neoadjuvant chemoradiotherapy, rectal cancer, proteomics

## Abstract

Locally advanced rectal cancer (LARC) presents a challenge in identifying molecular markers linked to the response to neoadjuvant chemoradiotherapy (nCRT). This study aimed to utilize a sensitive proteomic method, data-independent mass spectrometry (DIA-MS), to extensively analyze the LARC proteome, seeking individuals with favorable initial responses suitable for a watch-and-wait approach. This research addresses the unmet need to understand the response to treatment, potentially guiding personalized strategies for LARC patients. Post-treatment assessment included MRI scans and proctoscopy. This research involved 97 LARC patients treated with intense chemoradiotherapy, comprising radiation and chemotherapy. Out of 97 LARC included in this study, we selected 20 samples with the most different responses to nCRT for proteome profiling (responders vs. non-responders). This proteomic approach shows extensive proteome coverage in LARC samples. The analysis identified a significant number of proteins compared to a prior study. A total of 915 proteins exhibited differential expression between the two groups, with certain signaling pathways associated with response mechanisms, while top candidates had good predictive potential. Proteins encoded by genes *SMPDL3A*, *PCTP*, *LGMN*, *SYNJ2*, *NHLRC3*, *GLB1,* and *RAB43* showed high predictive potential of unfavorable treatment outcome, while *RPA2*, *SARNP*, *PCBP2*, *SF3B2*, *HNRNPF*, *RBBP4*, *MAGOHB*, *DUT*, *ERG28*, and *BUB3* were good predictive biomarkers of favorable treatment outcome. The identified proteins and related biological processes provide promising insights that could enhance the management and care of LARC patients.

## 1. Introduction

Colorectal cancer (CRC) is the third most common type of cancer worldwide, with almost two million newly diagnosed cases in 2020 [1]. High mortality rates place CRC in second place after lung cancer [1]. In Serbia, the situation is similar. According to data from the Institute of Public Health “Milan Jovanovic Batut” of the Republic of Serbia, CRC holds second place by incidence and mortality rates, with around 5000 new cases and 3000 deaths annually [2]. In the majority of cases, it is diagnosed in advanced stages where limited treatment options are available and survival is poor. Our group and others have invested efforts into profiling the diagnostic, prognostic, and predictive factors for CRC and anal cancer in an effort to provide better research strategies for treatment and overall management [3,4,5,6,7]. However, current early detection and screening programs, as well as treatment options, need further improvement on a global level. Rectum is the most distal part of the digestive tract, located between the sigmoid colon and anal canal. Colon and rectal cancers have been traditionally considered as a single disease entity, and rectal cancer (RC) represents around 35% of diagnosed CRC cases. Rectal cancer has distinct environmental and genetic risk factors that differentiate it from colon cancer [8]. Its incidence has been reported to increase in the 18- to 50-year-old age group, especially in younger adults [9].

Locally advanced rectal cancer (LARC) is the most diagnosed type of RC, which includes stage II (T3/4N0M0) and III (T1-4 N+ M0), according to the Union for International Cancer Control (UICC) [10]. The standard treatment for LARC is neoadjuvant chemoradiotherapy (nCRT) followed by radical surgery (total mesorectal excision). nCRT was established as the gold standard of LARC treatment after 2004 as a result of two studies, CAO/ARO/AIO-94 and EORTC 22921, which compared it to previously used adjuvant radiotherapy with or without chemotherapy. According to the CAO/ARO/AIO-94, neoadjuvant RT dramatically reduced the rates of local failure, while the 11-year follow-up update showed that the long-term overall survival rate was about 60% [11]. The EORTC 22921 trial showed that the use of chemotherapy with neoadjuvant radiation reduced local recurrence rates but had no effect on distant progression-free survival. Additionally, nCRT contributed to sphincter preservation and improved the patient’s quality of life [12]. However, only 20–30% of patients experience a complete clinical or pathological response to nCRT, while some patients will experience poor response or will have distant progression during nCRT [13,14]. Characterization of mechanisms of response to therapy and the search for predictive biomarkers to nCRT is an unmet need in LARC.

The watch-and-wait approach was introduced because of the need for close follow-up of LARC patients with complete clinical response to nCRT and allowed the extension of periods between neoadjuvant therapy and surgery, thus lowering morbidity related to surgery [15,16,17,18]. No biomarker has yet been validated in this setting. Patients with a favorable response to nCRT would be candidates for a less invasive surgical approach or would be enrolled in a watch-and-wait approach in the case of a complete clinical response (cCR). That would increase their quality of life and contribute to the overall reduction of treatment costs [18].

In this study, we aimed to perform in-depth proteomics characterization of preoperative LARC biopsy samples by employing data-independent acquisition mass spectrometry (DIA-MS) to unravel new tissue molecular features that might lead to different responses to nCRT.

## 2. Results

### 2.1. Study Design and Rationale

Neoadjuvant chemoradiotherapy is a standard treatment for locally advanced rectal cancer, but understanding the mechanism of response to therapy is still an unmet need. For that purpose, we examined our cohort of 97 LARC patients whose clinical profile was well characterized. Ninety-seven patients with LARC treated at the Institute for Oncology and Radiology of Serbia from 2018 to 2019 were included. Inclusion criteria comprised histopathologically confirmed LARC (T3-T4N0 or any T stage N+), a distant margin up to 12 cm from the anal verge, and ECOG performance status ≤ 2. Patients received long-course chemoradiotherapy with concurrent chemotherapy. Tumor response was assessed after surgery according to the classification by Mandard, and patients were divided into responders (TRG 1 and 2—complete and near complete response, respectively) and non-responders (TRG 3, 4, and 5—moderate, poor, and no response, respectively) based on pathohistological criteria. Our study involved the analysis of 20 patient samples exhibiting a range of responses to achieve a comprehensive understanding of the diverse molecular features potentially associated with response. The observed results will help us better understand the mechanisms behind the response, enabling the selection of protein candidates that can serve as predictive biomarkers for favorable or unfavorable responses. Patients expected to have a favorable response may be considered for a less invasive surgical approach or enrollment in a watch-and-wait strategy in case of complete clinical response (cCR). This approach can lead to good local control without the morbidity associated with radical surgery after neoadjuvant treatment, subsequently improving the quality of life in this group of patients. Conversely, non-responders may be candidates for intensified neoadjuvant treatment and earlier surgical intervention without delay following initial treatment, the introduction of targeted therapy, or other treatment adoptions.

### 2.2. Proteomic Comparison of Responders and Non-Responders

**Figure 1:**
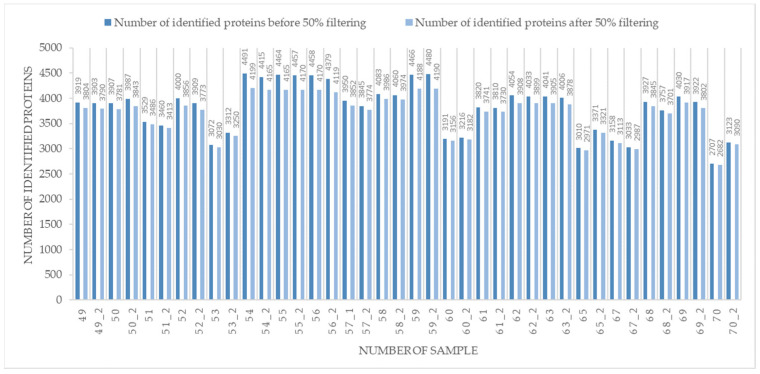
Number of unique proteins identified before and after 50% filtering.

**Figure 2:**
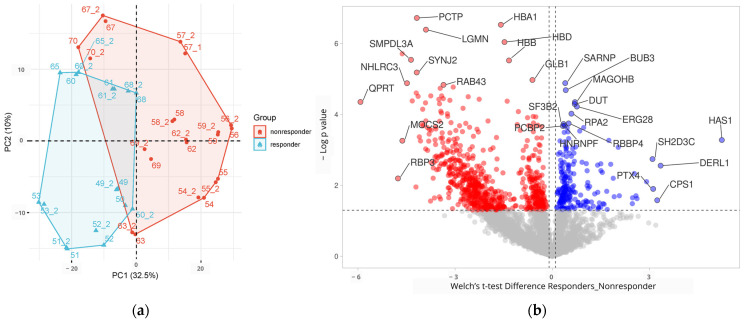
Principal Component Analysis (**a**) and a volcano plot (**b**) of differentially abundant proteins in terms of response to treatment. PCA (**a**) indicated that responders (blue) compared to non-responders (red) had significantly different proteomics profiles. Proteomics profiles of responders vs. non-responders were compared, and results were represented by volcano plot as −log10 (Welch *t*-test *p*-value) plotted against the difference (responders–non-responders). Genes upregulated in responders were colored blue, and those upregulated in non-responders were colored red. Top 10 with highest statistical difference are colored in both groups, and top 5 with highest Welch *t*-test difference.

The use of DIA-MS allowed the identification and quantification of more than 3000 proteins per sample Figure 1, a significant increase when compared to the 1000 proteins identified by DDA-MS in rectal cancer FFPE samples [19]. In total, 4269 proteins were identified in 20 rectal cancer FFPE samples (Table S1), while 1923 proteins were identified in all samples. After 50% filtering and log2 transformation, the Welch *t*-test was applied. The number of identified proteins before and after 50% is shown in Figure 1. Statistical analysis indicated 915 DEPs with significant differences (*p* < 0.05; S0 = 0.1) between responders and non-responders. When two groups were compared, DEPs included 700 proteins overexpressed in non-responders and 215 overexpressed in responders (Table S2 and Figure 2b). Using a more stringent statistical setting (*p* < 0.01; S0 = 0.1), 384 DEPs were found between responders and non-responders, 81 of which were upregulated in responders compared to non-responders, and 303 proteins with the opposite trend (Table S3).

Principal Component Analysis (PCA) (Figure 2a) shows the separation of the patients depending on their proteomic profile, indicating that responders had significantly different proteomics profiles than non-responders. On the PCA plot, we observe two samples that are outliers from the group of non-respondents (67 and 70) as well as sample 65 from the group of respondents. A potential reason for this behavior is the fact that these samples have a lower number of identified proteins compared to others, which may be associated with the lower quality of the sample and divergent proteomic profile. For comparison, a volcano plot (−log10(*p*-value) vs. Welch *t*-test difference) was created to graphically show the proteome changes between the two groups of samples (Figure 2b).

**Figure 3:**
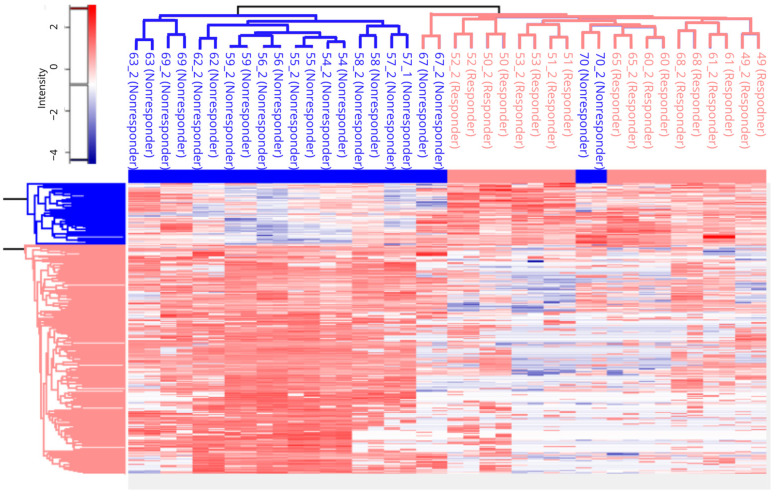
Hierarchical cluster analysis was performed on the proteomic data. The results indicate the existence of two clusters of patients based on proteomic analysis, which are highly correlated with the clinical response to therapy with two outliers (responders are written in blue, and non-responders are written in pink).

Hierarchical clustering analysis was performed on Z-score normalized data with stricter Welch’s *t*-test statistics (*p* < 0.01), while distance for analysis was performed using Euclidean distance (Figure 3). Hierarchical clustering analysis (Figure 3) revealed details on protein abundance among two groups of responders and non-responders; clustering also provided clear grouping results depending on the response, with samples 67 and 70 as an outlier. This sample had the lowest amount of detected proteins, which can be a possible reason for aberrant classification.

**Figure 4:**
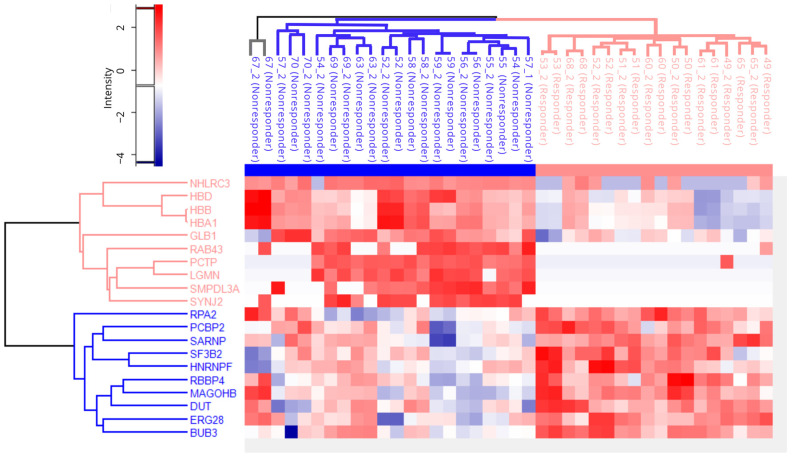
Hierarchical clustering analysis revealed details on the protein abundance of the top 20 candidates among two groups of responders and non-responders. Y axis represents the top 10 overexpressed DEPs in both the responder (pink) and non-responder (blue).

Furthermore, a hierarchical clustering analysis was performed for the top 10 proteins from the group of responders and non-responders based on the strength of statistical significance (Figure 4). Based on the obtained results, we conclude that the tissue proteomic profile differs significantly between patients and thus enables their clear classification. Proteins encoded by genes *SMPDL3A*, *PCTP*, *LGMN*, *SYNJ2*, *NHLRC3*, *GLB1,* and *RAB43* showed high predictive potential of unfavorable treatment outcome, while *RPA2*, *SARNP*, *PCBP2*, *SF3B2*, *HNRNPF*, *RBB4*, *MAGOHB*, *DUT*, *ERG28,* and *BUB3* were good predictive biomarker of favorable treatment outcome. Hemoglobin-related proteins (*HBB*, *HBA1,* and *HBD*) can be possible contaminants originating from FFPE tissue samples. The mentioned proteins are good candidates with great potential for validation on a larger group of patients, which would confirm their discriminatory potential.

### 2.3. Pathway Enrichment Analysis

**Figure 5:**
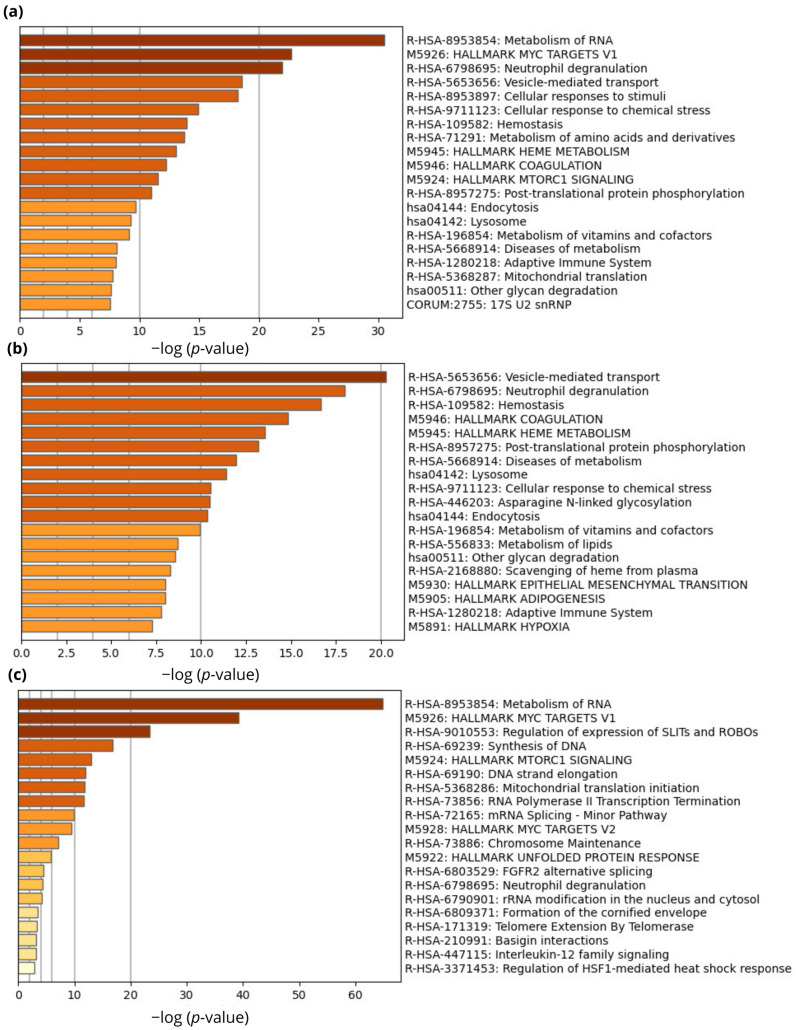
Enrichment pathway analysis of the differentially expressed proteins between responders and non-responders. Pathway enrichment analysis was conducted using the Metascape program. All statistically enriched terms were first identified by software, and then accumulative hypergeometric *p*-values and enrichment factors were computed and used for filtering. The remaining relevant terms were subsequently hierarchically clustered into a tree based on the similarity of their gene memberships as measured by Kappa statistics. The obtained results were considered and represented based on biological relevance with respect to rectal cancer biology. KEGG, Reactome, Corum, and Hallmark databases were used for pathways enrichment analysis. (**a**) Bar graph of enriched signaling pathways of the differentially expressed proteins (915 DEPSs) (colored by *p*-values). (**b**) Bar graph of enriched signaling pathways of the differentially expressed proteins overexpressed in non-responder group (700 DEPs) (colored by *p*-values). (**c**) Bar graph of enriched signaling pathways of the differentially expressed proteins overexpressed in responder group (215 DEPs) (colored by *p*-values).

Enrichment pathway analysis was performed on proteins that were significantly differentially expressed (*p* < 0.05; S0 = 0.1). Initially, all proteins were chosen regardless of grouping with the goal of better understanding the signaling pathways involved in treatment response (Figure 5a). Further enrichment analysis was carried out on two groups (responders and non-responders) independently in an attempt to explain discrepancies in treatment response. The findings were analyzed and represented based on their biological significance to RC biology. To keep the analysis output concise, only the leading terms of each pathway are shown. Results indicated that some of the leading signaling pathways that correlate with response to nCRT in patients with LARC include the metabolism of RNA, MYC targets, neutrophil degranulation, cellular transport, and response to stimuli. The responder group was characterized by signaling pathways related to cell cycle signaling (metabolism of RNA, synthesis of DNA, DNA strand elongation, mitochondrial translation initiation, chromosome maintenance), as well as MYC targets scores, regulation of expression of SLITs and ROBOs, mTOR1 signaling pathway, and unfolded protein response (Figure 5c). The non-responder group was characterized by signaling pathways related to vesicle-mediated transport, neutrophil degranulation, hemostasis, coagulation, heme metabolism, post-translational modifications, as well as the metabolism of vitamins, cofactors, and lipids. Signaling pathways related to epithelial–mesenchymal transition and hypoxia, which have been associated with an increased risk of metastasis, were also found to be important in non-responders (Figure 5b).

### 2.4. STRING In Silico Analysis

Data obtained reveal several protein-rich groups with several members having high levels of interactions in the responder and non-responder group (PPI enrichment *p*-value: <1.0 × 10^−16^), indicating that proteins interact with one another more frequently than would be predicted by a randomly selected group of proteins from the genome with the same size and degree distribution [20]. After Cytoscape analysis using MCODE extension, six clusters were detected. We can conclude that there is a strong interaction between proteins involved in information RNA processing and genes whose protein products participate in transcription, especially when it comes to pre-mRNA processing (Figure S1a) and factors involved in the process of alternative splicing (Figure S1b). A high degree of interaction is also associated with proteins that participate in the formation of snRNA molecules (Figure S1b). Another group of proteins that are clearly distinguished based on STRING analysis are the ribosomal proteins of the RPL family (Figure S1e) and MRPL family (Figure S1f), as well as PA2G4, which represents the proliferation factor (Figure S1e). All the mentioned groups of proteins are characterized by a high mutual connection. Another group of proteins includes factors involved in the DNA replication process as well as factors for the organization of the proteasomal system (Figure S1c,d). On the other hand, the group of DEPs overrepresented in the non-responder group is characterized by a much larger number of proteins that are less closely related to each other. One of the detected clusters included proteins that control cell death, proliferation, and signal transduction (Figure S2a), as well as proteins involved in lipid metabolism (Figure S2b). Cluster 3 included proteins that are related to the mitochondrial electron transport chain (Figure S2c), while cluster 4 included proteins related to retrograde electron transport (Figure S2d), followed by purine and pyrimidine catabolism (Figure S2d) and the HLA class of proteins included in antigen processing and presentation (Figure S2e).

### 2.5. Shortlisting of Potential Biomarkers Based on Transcriptomics Data

**Figure 6:**
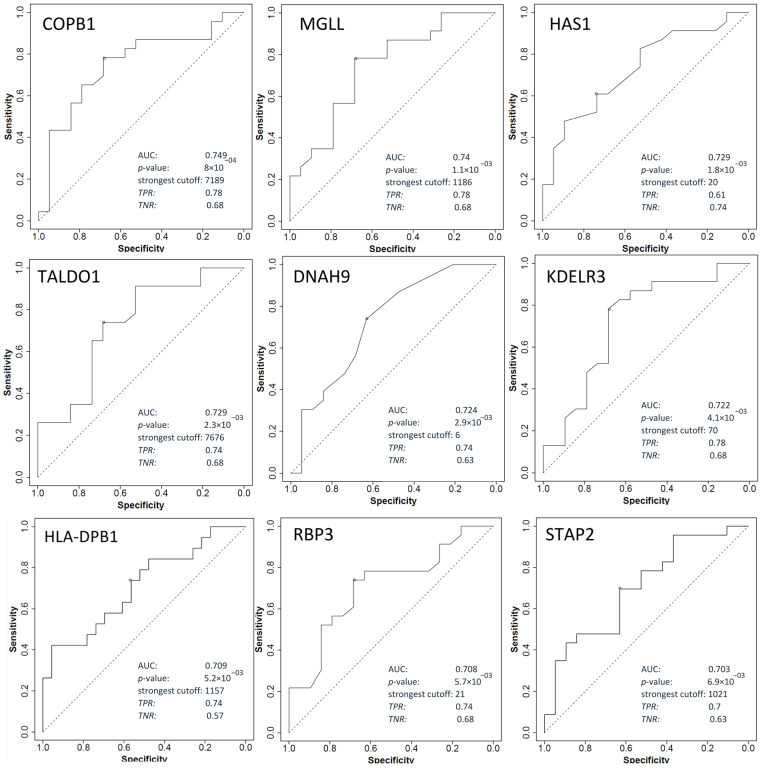
ROC plot of genes overexpressed in non-responder group.

**Figure 7:**
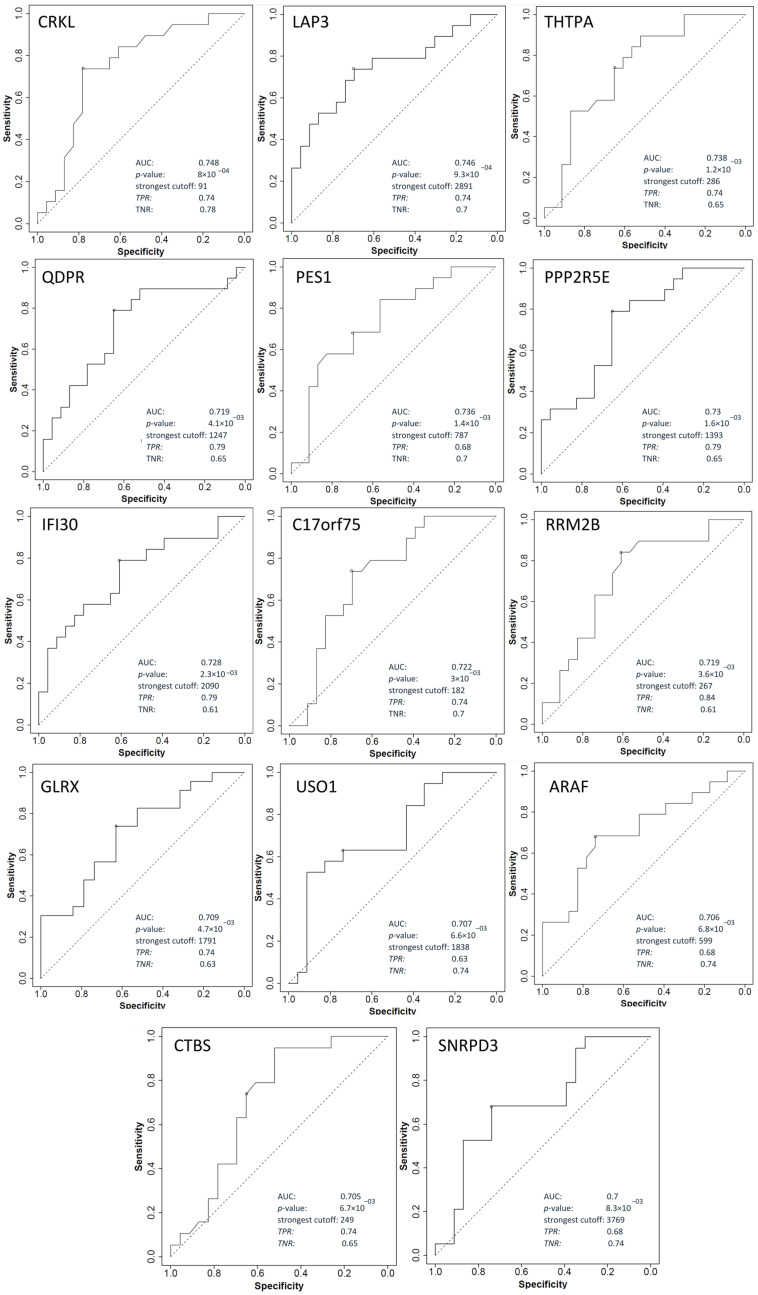
ROC plots of genes overexpressed in responder group.

The proteomics results obtained were further examined to discover promising predictive biomarkers of response to neoadjuvant chemoradiotherapy in patients with LARC. The differential expression of proteins identified in our study was confirmed in transcriptomics datasets. The ROC curve was considered to assess the performance of predictive biomarkers for response to chemoradiotherapy. For this purpose, DEPs obtained after DIA-MS/MS were analyzed using ROCplotter software (https://www.rocplot.org, accessed on 1 May 2022), and genes with AUC > 0.7, ROC *p*-value < 0.05, and Mann–Whitney *p*-value < 0.05 were categorized as promising biomarkers. Out of a total of 915 DEPs, 23 genes met all three criteria. The responder group had the following proteins whose expression was also confirmed at the mRNA level: *CRKL*, *LAP3*, *THTPA*, *PES1*, *PPP2R5E*, *IFI30*, *C17orf75*, *QDPR*, *RRM2B*, *USO1*, *GLRX ARAF*, *CTBS,* and *SNRPD3*. Moreover, the non-responder group had the following proteins, *COPB1*, *MGLL*, *HAS1*, *TALDO1*, *DNAH9*, *KDELR3*, *HLA-DPB1*, *RBP3* and *STAP2*, as presented in Table 1 and Table 2. ROC curves and their discriminatory potential for the mentioned genes are shown in Figure 6 and Figure 7. Linking proteomics with transcriptomics data can lead to the discovery of promising rectal cancer biomarkers that are easy, cost-effective, and fast to detect.

### 2.6. Search for Drug Targets

In order to search for drug targets among proteins that are differentially overexpressed in the group of non-responders, GeneCards and DrugBank databases were searched. Some of the proteins that are differentially overexpressed in the group of non-responders versus responders are drug targets used in the treatment of some other pathological conditions including *QPRT*, *CLCA4*, *ATG4B*, and *PTGS2* (Table 3) [22,23]. The question of the discriminatory effect of gene expression is raised, as well as whether the use of these drugs can be used as a part of initial treatment and would lead to a better response to therapy by treating patients with locally advanced rectal cancer together with standard neoadjuvant chemoradiotherapy.

## 3. Discussion

Understanding the molecular features associated with response to neoadjuvant chemoradiotherapy is an unmet clinical need in LARC. The DIA-MS offers unprecedented proteome coverage for FFPE samples. DIA-MS enabled the in-depth study of the proteome from FFPE tissue samples, which represented a major challenge because of damage caused by the fixation protocol [24]. By exploring the dynamic phenotypic characteristics of tumor cells before therapy and tumor response to therapy, DIA-MS allows us to characterize response mechanisms and thus enable patient monitoring and more effective treatment. FFPE samples are routinely used for DDA-MS analysis, and the use of FFPE samples for the DIA-MS method is increasing [25]. In contrast to fresh frozen (FF) tissue, FFPE samples undergo protein cross-linking during standard preservation protocol, and due to that, it is challenging to analyze native proteins. Comparing the results obtained when it comes to FF tissue versus FFPE samples indicates a high correlation between the results, which makes FFPE samples a good alternative to FF samples [26,27]. On the other side, FFPE samples are more suitable for retrospective studies and are easily accessed during everyday clinical routines [25]. Due to technical limitations, results obtained in the previous studies showed a restricted number of identified proteins, while DIA-MS/MS offered an in-depth characterization of rectal cancer tissue, enabling molecular characterization and profiling of the response [19,28,29]. Our study included analysis of discrete and well-characterized clinical samples of rectal cancer in order to identify the maximum number of different molecular features potentially associated with response.

In total, 4269 proteins were identified in 20 rectal cancer FFPE samples. Principal Component Analysis (PCA) indicated that responders had a significantly different proteomics profile than non-responders. Statistical analyses comparing the two groups resulted in the identification of 915 differentially expressed proteins (215 in responders and 700 in non-responders) (*p* < 0.05).

The therapy approach used for LARC includes radiotherapy in combination with 5-fluorouracil-based chemotherapy followed by surgery. Radiotherapy primarily exerts its effects by damaging DNA through the generation of molecular fragments like free radicals and excited molecules. Cells are most sensitive to radiation during mitosis and the early G1 phase, while they are most resistant during the S phase of the cell cycle [30]. The abovementioned effects are first reflected in the cell cycle, and the overcoming of toxic effects can be carried out through the reparation of the resulting damage. 5-fluorouracil is a standard chemotherapeutic drug (antimetabolite) that is metabolized by the liver. Within cells, 5-FU undergoes metabolic conversion, resulting in three active metabolites: 5-FdUMP and 5-FdUTP, which damage DNA, and 5-FUTP, which integrates into RNA, exerting an antiproliferative effect. Our finding confirmed the significance of DNA metabolism and related signaling pathways in treatment response. Patients with a poor response exhibit deregulated pathways related to DNA strand elongation and synthesis.

Based on hierarchical clustering analysis, we conclude that the tissue proteomic profile differs significantly between patients, which enables their clear classification (Figure 4). Additionally, some of those proteins have been shown to be included in the development and progress of many other types of cancers, but none of them were investigated in terms of rectal cancer therapy resistance. All of the top 10 proteins from both groups showed high predictive potential. It has been shown that protein legumain (*LGMN*) is overexpressed in breast, prostate, and liver cancer and that its role is significant in cancer development, progression, and invasion [31,32]. This protein was also shown to be significantly associated with the development of peritoneal metastases. In our study, this protein was shown to be a promising predictive biomarker of poor response to therapy. This protein is a member of several pathways shown to be significant in response to nCRT, including mTOR, coagulation, adaptive immune system, and lipid metabolism. Pathway mTORC1 was altered in the group of good responders. Apart from *LGMN,* there is BUB3, which is shown to be overexpressed in the responder group and included in the mTOR signaling pathway as well. Analysis of DEPs provided a potential scenario that included the downregulation of genes related to the mTORC1 pathway in responders or overexpression of mTORC1-related genes in non-responders. Considering available data, both scenarios will lead to poor response to treatment. It has been demonstrated that treatment resistance in a variety of cancer types is correlated with the stimulation of mTOR signaling pathways [33,34]. Glycolysis, glycoprotein and lipid synthesis, mitochondrial and lysosome biogenesis, and metabolic balance all depend on mTORC1. Translation is directly impacted by the regulation of numerous transcriptional factors. It regulates the production of nucleotides and the metabolism of glucose through proteins in the metabolic pathway. Additionally, mTORC1 regulates the assembly of the proteasome and autophagy [35]. Glucose metabolism, apoptosis, cell migration, cytoskeletal reorganization, and cell proliferation are regulated by mTORC2 [36,37]. According to our results, signaling pathways related to protein and lipid synthesis, glycolysis, mitochondrial biogenesis, and lysosome biogenesis correlate with poor response to nCRT, which is in compliance with previous findings. An in-depth characterization of mTORC1 signaling pathways is needed to shed light on the exact biochemical mechanism that leads to good/poor responses to treatment.

Our results indicated that the immune response might play an important role in predicting response to therapy. Signaling pathways associated with IL-12 were found to correlate with a good response to therapy, while signaling pathways related to the adaptive immune system were related to a poor response. Heeran et al. highlighted the association of inflammation with obesity status in rectal cancer patients in terms of lowering the level of inflammatory factors released from TME [38]. Our data correlated adipogenesis with poor response treatment. As increased adipose tissue synthesis is directly associated with a rise in body weight, it could also result in reduced therapeutic response. A study by Lee et al. has shown that obesity represents an independent predictor of cCR, which contradicts our results [39]. In some studies, no clear correlation was found between obesity and rectal cancer treatment outcomes [40]. Next to it, Synaptojanin 2 (*SYNJ2*) was shown to be a good marker of poor prognosis in lung cancer. This protein is included in signaling pathways altered in the non-responder group, including vesicle-mediated transport and lipids. When it comes to predictive markers of a good response, none of the presented proteins were investigated in terms of predictive potential. It was shown that RB-binding protein 4 (*RBBP4*) is associated with a poor prognosis of colon cancer, while our results suggest a favorable effect. This protein is part of two signaling pathways that are alerted in good response. Slit–Robo signaling plays an important role in angiogenesis. The vertebrate Robo4 gene, which has been associated with regulating angiogenesis and blood artery permeability, has highly specific endothelial cell expression [41,42]. In our cohort, it was shown that the regulation of SLITs and ROBOs is highly correlated with a good response to nCRT. Slit/Robo signaling has both pro- and anti-angiogenic functions; therefore, its effect on angiogenesis depends on the environment. It has been demonstrated that Slit2 stimulates angiogenesis through the Robo1 receptor but that it also inhibits endothelial migration through the Robo4 receptor, which aids in a positive therapeutic response. It was also reported that Split3 promotes angiogenesis [43]. Apart from *SYNJ2*, *MAGOHB* and DNA-directed RNA polymerase I subunit RPA2 were shown to be overexpressed in responder group. In terms of good response to treatment and favorable outcomes, inhibition of endothelial migration and angiogenesis is preferred. Based on the results of research previously conducted by our group, we found that hematological parameters, including neutrophil-to-monocyte ratio, initial basophil, eosinophil, and monocyte counts, are significantly different between the responder and non-responder groups. According to MRI findings, we realized that non-responders are more often presented with extramural vascular invasion [44]. In support of all of those observations, our results indicated the importance of epithelial–mesenchymal transition in patients with a poor response to therapy. Hypoxia has also been linked with neutrophil degranulation. In hypoxic conditions, degranulation occurs, and released factors affect tumor progression [45]. According to our results, the neutrophil degranulation pathway was affected in both the responder and non-responder groups, which highlights its importance in this process and warrants further functional studies. And some proteins of this pathway that showed good predictive potential are coded by gene *HNRNPF* in the responder group and NHL repeat-containing protein 3 (*NHLRC3*) and Beta-galactosidase (*GLB1*) in the non-responder group.

In rectal cancer patients receiving neoadjuvant CRT, it was shown that high expression of FGFR2 was associated with an advanced tumor stage, a poor treatment response, and lower survival [46]. The DIA-MS approach indicated the importance of FGFR2 alternative splicing in good response to treatment, and the protein encoded by gene *HNRNPF* was shown to have good predictive potential, which implies that exploring its variants might be useful for the prediction of a good outcome.

The highest correlation with poor response to treatment was shown for signaling pathways related to vesicle-mediated transport and endocytosis, while proteins encoded by genes *LGMN*, *GLB1*, and *SYNJ2* and Ras-related protein Rab-43 (*RAB43*) are part of listed signaling pathways and good predictors of poor response. Proteins and other cargo must be carried through the cell via a cellular transport mechanism in which the transported materials are conveyed in membrane-bound vesicles. The vesicle lumen or the vesicle membrane is where transported compounds are contained [47]. By carrying biomolecules (proteins, lipids, deoxyribonucleic acid, and ribonucleic acid) throughout the tumor microenvironment, exosomes released from cell membrane play a crucial role in tumor proliferation, differentiation, metastasis, and resistance to chemotherapy and radiation [48]. The results obtained in this study indicated a great potential for exploring intercellular communication in the tumor microenvironment as well as in the tumor when profiling response to therapy. The synergistic effect of these inter-relations has not yet been clarified and would also be validated by our group in future functional studies.

Deoxyuridine triphosphatase (*DUT*) is associated with a shorter DFS in patients with CRC [49]. This protein is important for DNA strand elongation and unfolded protein response and is an MYC target. In addition to *DUT*, *SF3B2* and BUB3 were shown to be overexpressed in responder group. Signaling pathways related to MYC targets have also been found to be significant for the good response to nCRT in our LARC cohort. MYC is an oncogene and transcription factor that regulates cell-cycle-related signaling pathways, supporting their crucial effect in response to treatment [50]. Signaling pathways, which include chromosome maintenance and telomere extension by telomerase, were found to be affected in good response. This observation suggested that changes at the chromosome level can lead to a good response to treatment and increase the efficiency of RT. Short telomeres can lead to chromosomal instability and the formation of cancer, while on the other hand, long telomeres, due to their length, can undergo a higher number of divisions and thus increase the probability of transformation of a normal cell into a malignant cell, this phenomenon called telomere length paradox [51]. When it comes to the response to therapy, the correlation between telomere length and cancer treatment is not fully clarified [52]. Additionally, mTOR, a key regulator of cell growth and division in healthy conditions, can be inappropriately activated in tumor cells and thus promote tumor cell growth, metastasis, and invasion of fresh, healthy tissues [53]. Cytokines and growth factors are released during immunological response against harm that CRT may cause, and they play a big part in the generation of ROS, including superoxide, hydrogen peroxide, and nitrogen (II)-oxide [54]. Interleukins (IL-2, IL-12) have been found in preclinical studies that might help to modulate the antitumor response and radiosensitize cells [55]. IL-12 achieves its antitumor activity by promoting the immune response via the activation of natural killer cells and cytotoxic T cells and exerting an anti-angiogenic effect [56,57]. In a study performed by Heeran et al., increased levels of IL-12 were detected in the blood of LARC patients compared to healthy individuals. This suggests the potential for promoting an immune response that may lead to improved treatment outcomes [38]. Our study showed that the protein encoded by the gene *HNRNPF* has predictive potential for good treatment outcomes.

The data in the literature from the past several decades identified altered glycosylation as a sign of malignancy. It was shown that glycosylation acts as a mediator of the inflammatory response [58]. In gliomas and laryngeal carcinoma, a correlation between glycosylation and radioresistance has already been demonstrated [59]. According to our results, glycan degradation and asparagine N-linked glycosylation might play an important role in the poor response of LARC patients to nCRT, proteins encoded by genes *PCTP* and *GLB1* shown to have high potential in the prediction of poor response to treatment.

Linking the transcriptomic and proteomic profiles of the cell is an important parameter for understanding the molecular basis of the response to therapies. The software ROC plotter (www.rocplot.org, accessed on 3 May 2022) showed a low correlation between gene expression and the proteome profile of the tested samples. Out of 915 differentially expressed proteins, only 23 showed promising discriminatory potential when it comes to gene expression. It should be kept in mind that the transcriptomics data were obtained from samples of different demographics and ethnic origins; therefore, further validation on patient samples within our cohort should be performed. Genes that have shown a transcriptional discriminatory potential for predicting a good response have also not been investigated in rectal carcinoma so far. Published results indicate that *PPP2R5E*, *PES1*, *RRM2B*, *GLRX*, and *CRKL* are important in the prognosis and development of colorectal cancer [60,61,62,63,64,65]. When it comes to the prediction of poor response, some of the aforementioned genes showed a poor prognostic potential risk for the development of CRC. The *HLA-DPB1* gene has been shown to be a good predictive marker of response to nCRT in patients with rectal cancer [66]. These results indicate that there is great potential in examining the level of expression of the mentioned genes. Exploiting this potential would lead to rapid, inexpensive, and easy methods for predicting response to therapy in patients with LARC. The translation of research from protein to gene expression and confirmation of the obtained candidates would enable a more cost-effective approach and, thus, a more efficient selection of patients when it comes to predicting the response to neoadjuvant chemoradiotherapy. Gene expression analysis shows high sensitivity when it comes to FFPE tissue analysis and would be more likely to be performed during everyday practice. At the same time, further analysis of the gene expression profile and correlation with a proteomic profile of the tissue would enable a more detailed investigation of the mechanism behind response treatment, which is still unclear.

Based on all obtained results, we conclude that there is a statistically significant difference between the proteomic profiles of LARC patients who respond well and those who respond poorly to nCRT. As all patients require surgery after nCRT per current guidelines, profiling of adequate biomarkers of response is a pressing matter. Further validation of target signaling pathways detected in this study that might have an effect on the response to nCRT is planned on a prospective cohort of LARC patients at the Institute for Oncology and Radiology of Serbia to ensure more efficient and cost-effective treatment of patients while maximizing their quality of life.

## 4. Materials and Methods

### 4.1. Patient Cohort Characteristics and Treatment

A total of 97 LARC patients treated at the Institute for Oncology and Radiology of Serbia in the period of 2018–2019 were included in this study. The inclusion criteria were histopathologically verified adenocarcinoma of the rectum, with a distant margin up to 12 cm from the anal verge by rigid proctoscopy, ECOG performance status ≤ 2. LARC was defined as T3–T4N0 or any T stage N+, according to clinical and histological criteria of the 8th edition of the TNM classification of malignant tumors [67]. Pretreatment evaluation included an abdominal and pelvic MRI scan and a computed tomography (CT) scan or X-ray of the chest. All patients were treated with long-course chemoradiotherapy (CRT). Radiotherapy (RT) was delivered with a total dose of 50.4 Gy in 28 fractions (conventionally fractioned 1.8 Gy/fr), using the technique with 3 or 4 radiation areas (all areas as recommended by the International Committee of Radiation Units and Measurements (ICRU, 50/62 per day) [68,69]. Concomitant chemotherapy was initiated on the first day of RT and administered during the first and fifth weeks of RT. The chemotherapy regimen included 5-FU (350 mg/m^2^ daily) and Leucovorin (25 mg/m^2^ daily). A complete patient medical database has been prepared from official records.

Patients were assessed for tumor response between the 6th and 8th weeks after CRT completion with pelvic MRI scan and rigid proctoscopy. The pathohistological response after surgery was assessed according to tumor regression grading (TRG) categories by Mandard [70]. According to the TRG status, the patients were divided into two groups: responders (TRG 1–2) and non-responders (TRG 3–5). Our study included analysis of extreme candidates in order to achieve the maximum range of different molecular features potentially associated with response. Twenty-four formalin-fixed paraffin-embedded (FFPE) biopsy samples were taken at the moment of disease diagnosis and were collected and used for proteomic analysis. After the quality control check, four samples were excluded from further analysis, and finally, twenty samples were processed (9 responders and 11 non-responders). Characteristics of the study cohort are shown in Table 4.

The project was approved by the Ethics Committee of the Institute for Oncology and Radiology of Serbia (approval No. 2211-01 from 11 June 2020), and all patients signed informed consent. All experiments have been performed in accordance with the Helsinki Declaration of 1975, as revised in 2013.

#### Protein Extraction from FFPE Tissue Samples

From each of the 20 FFPE LARC samples (9 responders and 11 non-responders), 10 sections 15–20 μm thick were cut using a microtome and transferred to 2 mL Eppendorf tubes. From all sections, 3 were selected that contained the largest amount of tissue. Samples were deparaffinized using Xylene in two steps of 5 min and 1 min successively. The samples were centrifuged, and in the next step, the pellet tissue was rehydrated using several dilutions of ethanol and finally washed with double distilled water. The tissue pellet was air-dried and dissolved in FASP protein extraction buffer (100 mM Tris-HCl pH 7.6, SDS 4%, 100 mM 1,4-Dithioerythtriol (DTE)). The tissue was homogenized, while the disintegration of the cell membrane was achieved by sonication (three cycles of 5 s with 36% power). The samples were heated at 90 °C for 1 h to extract the cellular proteins into the solution. The supernatant containing extracted proteins was transferred to a new Eppendorf tube, and an appropriate amount of ammonium bicarbonate (ABC) buffer was added. The sample was concentrated using a 3 kDa cut-off Amicon filter.

### 4.2. Protein Digestion and Preparation for LC-MS/MS Analysis

The total volume of concentrated proteins was added on SDS PAGE (5% stacking gel, 12% separating gel). Preparative SDS PAGE was performed, and the gel was fixed, washed, and stained with Coomassie colloidal dye (File S1). Protein bands were cut from the gel for each sample separately, chopped, and transferred to Eppendorf tubes. The strips were decolorized with a solution of 40% Acetonitrile and 50 mM NH_4_HCO_3_ until parts of the gel became completely transparent. The samples were reduced with 10 mM DTE in 100 mM NH_4_HCO_3_ and alkylated 10 mg/mL Iodoacetamide in 100 mM NH_4_HCO_3_ and then washed with 100 mM NH_4_HCO_3_, destain solution, and water, respectively. The samples were dried in a speed vac until transparent crystals formed. Each sample was treated with trypsin solution, which enables the cutting of the polypeptides after the amino acids lysine and arginine. The peptides formed were extracted with NH_4_HCO_3_ solution followed by incubation in a 1:1 solution of 10% formic acid and Acetonitrile. The peptide solution was purified using PVDF filters (Merck Millipore, Darmstadt, Germany). The samples were dried in a Speedvac and prepared for further processing.

### 4.3. LC-MS/MS Analysis

Samples were run in two technical replicates on a liquid chromatography–tandem mass spectrometry (LC-MS/MS) setup consisting of an Ultimate 3000 RSLC online with a Thermo Q Exactive HF-X Orbitrap mass spectrometer. Peptide solutions were directly injected and separated on a 25 cm long analytical C18 column (PepSep, 1.9 μm beads, 75 µm ID) using a gradient of 7% to 36% Buffer B (0.1% formic acid in 80% Acetonitrile) for 70 min, followed by an increase to 95% in 5 min, a second increase to 99% in 0.5 min, and then kept constant for equilibration for 14.5 min. A full MS was acquired in profile mode using a Q Exactive HF-X Hybrid Quadropole-Orbitrap mass spectrometer, operating in the scan range of 375–1400 *m*/*z* using 120 K resolving power with an AGC of 3 × 10^6^ and max IT of 60 ms. Data-independent analysis followed, using 8 Th windows (39 loop counts) with 15 K resolving power with an AGC of 3 × 10^5^, max IT of 22 ms, and a normalized collision energy (NCE) of 26. The mass spectrometry proteomics data have been deposited to the ProteomeXchange Consortium via the PRIDE [71,72] partner repository with the dataset identifier PXD040451.

### 4.4. MS Data Analysis

Orbitrap raw data were analyzed in DIA-NN 1.8 (Data-Independent Acquisition by Neural Networks) by searching against the reviewed Human Uniprot database (retrieved 4/21) in the library free mode of the software, allowing up to two tryptic missed cleavages. Human Uniprot Database includes 27,246 proteins and 21,442 genes with 10,241,864 precursors generated. A spectral library was created from the DIA runs and used to reanalyze them. Parameters regarding peak generation and analysis are defined in the DIA-NN algorithm. DIA-NN default settings have been used with oxidation of methionine residues and acetylation of the protein N-termini set as variable modifications and carbamidomethylation of cysteine residues as fixed modification. N-terminal methionine excision was also enabled. A maximum number of variable modifications is set to 3. Both ends are fully tryptic, allowing up to two tryptic missed cleavages. The match between runs feature was used for all analyses, and the output (precursor) was filtered at 0.01 FDR. Retention time alignment is performed in DIA-NN. Correction for the mass accuracy is performed for each sample in DIA-NN automatically. Filtering of the quality is based on the false discovery rate of 0.01 at peptide and protein levels. Finally, the protein inference was performed on the level of genes using only proteotypic peptides. The analysis was set for at least one unique peptide per protein. The generated results were processed statistically and visualized in the Perseus software v1.6.15.0 (Max Planck Institute of Biochemistry, Munich, Germany) and GraphPad Prism 8.0.1. With the help of DIA-MS and DIA-NN processing, we identified 5756 groups of proteins, or 4875 unique proteins, that were used for subsequent analysis. Raw data were filtered based on a minimum of 50% valid values in at least one group of responder/non-responder and log2 transformed. After initial processing, non-human genes were excluded from further consideration, while missing values were replaced with imputed values that correspond to the limit of detection (LOD). The modified Student’s *t*-test, known as Welch’s Test for Unequal Variances, is used. In general, for samples with unequal variance, the adjusted degrees of freedom tend to increase the test power. Differentially expressed proteins (DEPs) were classified as proteins with *p* < 0.05 (Unequal Welch *t*-test with S0 cut off 0.1). Proteins with a Welch t-difference above 0 were classified as overexpressed in responders compared to non-responders, while proteins with a Welch *t*-test difference lower than 0 were classified as overexpressed in non-responders compared to responders. Visualization of the obtained results was performed using PCA, volcano plot, and hierarchical clustering. PCA was plotted by https://www.bioinformatics.com.cn/en accessed on 15 September 2023, a free online platform for data analysis and visualization, while volcano plots were plotted using https://huygens.science.uva.nl/VolcaNoseR/ accessed on 15 September 2023.

### 4.5. Pathway Enrichment Analysis

To understand the mechanism of response to treatment, pathway enrichment analysis was performed on DEPs between responders and non-responders using Metascape software (MSBio v3.5.20220422). Enrichment analysis parameters were set on a minimum of three genes overlapping between pathways and the input lists. Kyoto Encyclopedia of Genes and Genomes (KEGG) and Reactome and Hallmark (MSigDB) ontologies were used for correlation. Only statistically significant pathways (*p*-value ≤ 0.05 and minimum enrichment score above 1.5) were taken into account. The obtained results were considered and represented based on biological relevance with respect to RC biology. As a result, the leading term from each group is provided for simplification.

### 4.6. STRING In Silico Analysis

The STRING analysis network of DEPs overrepresented in responder/non-responder group was built based on the highest confidence (0.9) evidence from experimental interaction data, co-expression data, gene fusions, gene co-occurrence, gene neighborhood, and predictive and knowledge text mining. For easier data processing of 700 DEPs in non-responder group, disconnected nodes in the network were not presented. The analysis was performed using STRING v.11.5 and corresponding images and data downloaded in the original form with statistical significance set at *p* < 0.05 [20].

### 4.7. Shortlisting of Potential Biomarkers

Shortlisting of potential biomarkers was performed using the ROCplotter (www.rocplot.org, accessed on 3 May 2022), an online tool that uses the transcriptome data of a large set of rectal cancer patients (N = 284) to find gene expression-based predictive biomarkers. A single database was created by combining published gene expression data from accessible datasets with treatment information. Receiver operating characteristic (ROC) curve analysis was performed to assess the predictive accuracy of each gene [73,74]. The observed cohort included 42 patients treated with 5-fluorouracil and radiotherapy and was categorized into responders (N = 23) and non-responders (N = 19) according to the Response Evaluation Criteria in Solid Tumors (RECIST) criteria. Using a score method devised to assess each probe set for specificity, coverage, and degradation resistance, the optimal microarray probe set to represent a gene was chosen using the JetSet tool [75]. ROC curve with *p*-value < 0.05 was considered to evaluate the prediction ability of genes that showed a significant difference between the two groups.

## 5. Conclusions

Data-independent acquisition mass spectrometry (DIA-MS) analysis of FFPE LARC samples offered unprecedented in-depth proteomics characterization. Proteins encoded by genes *SMPDL3A*, *PCTP*, *LGMN*, *SYNJ2*, *NHLRC3*, *GLB1*, and *RAB43* showed high predictive potential of unfavorable treatment outcome, while *RPA2*, *SARNP*, *PCBP2*, *SF3B2*, *HNRNPF*, *RBBP4*, *MAGOHB*, *DUT*, *ERG28*, and *BUB3* were good predictive biomarkers of favorable treatment outcome. These newly identified molecular features associated with response to nCRT might prove useful for the construction of predictive panels to improve the management and care of LARC patients.

## Figures and Tables

**Table 1:** Shortlisted DEPs with characteristics of promising biomarkers enriched in the responder group compared to the non-responder group. Genes with an ROC *p*-value less than 0.05, an AUC greater than 0.7, and a Mann–Whitney *p*-value less than 0.05 were characterized as promising biomarkers.

Genes	Gene Name	Biological Function	FOLD Change	Mann–Whitney Test *p*-Value	AUC	ROC *p*-Value
***CRKL***	CRK-like Proto-Oncogene	Activate the RAS and JUN kinase signaling pathways and transform fibroblasts in a RAS-dependent fashion	1.4	0.0063	0.748	8.00 × 10^−4^
***LAP3***	Leucine Aminopeptidase 3	Predicted to enable peptidase activity; involved in proteolysis.	1.5	0.0059	0.746	9.30 × 10^−4^
***THTPA***	Thiamine Triphosphatase	Encodes an enzyme that catalyzes the biosynthesis of thiamine disphophate (vitamin B1) by hydrolysis of thiamine triphosphate	1.4	0.0089	0.738	1.20 × 10^−3^
***PES1***	Pescadillo Ribosomal Biogenesis Factor 1	Encodes a nuclear protein that contains a breast-cancer-associated gene 1 (BRCA1) C-terminal interaction domain	1.2	0.0096	0.736	1.40 × 10^−3^
***PPP2R5E***	Protein Phosphatase 2 Regulatory Subunit B’Epsilon	Protein phosphatase 2A is implicated in the negative control of cell growth and division	1.2	0.01	0.73	1.60 × 10^−3^
***IFI30***	IFI30 Lysosomal Thiol Reductase	This enzyme has an important role in MHC class II-restricted antigen processing	1.5	0.011	0.728	2.30 × 10^−3^
***C17orf75***	Chromosome 17 Open Reading Frame 75	Involved in intracellular protein transport and vesicle tethering to Golgi	1.3	0.015	0.722	3.00 × 10^−3^
***QDPR***	Quinoid Dihydropteridine Reductase	This gene encodes the enzyme dihydropteridine reductase, which catalyzes the NADH-mediated reduction of quinonoid dihydrobiopterin	1.3	0.013	0.719	4.10 × 10^−3^
***RRM2B***	Ribonucleotide Reductase Regulatory TP53 Inducible Subunit M2B	This heterotetrameric enzyme catalyzes the conversion of ribonucleoside diphosphates to deoxyribonucleoside diphosphates	1.3	0.016	0.719	3.60 × 10^−3^
***GLRX***	Glutaredoxin	This enzyme highly contributes to the antioxidant defense system	1.7	0.02	0.709	4.70 × 10^−3^
***USO1***	USO1 Vesicle Transport Factor	Peripheral membrane protein that recycles between the cytosol and the Golgi apparatus during interphase	1.2	0.022	0.707	6.60 × 10^−3^
***ARAF***	A-Raf Proto-Oncogene, Serine/Threonine Kinase	Involved in negative regulation of apoptotic process, regulation of TOR signaling, and regulation of cellular protein metabolic process	1.2	0.024	0.706	6.80 × 10^−3^
***CTBS***	Chitobiase	Lysosomal glycosidase is involved in degradation of asparagine-linked oligosaccharides on glycoproteins	1.3	0.024	0.705	6.70 × 10^−3^
***SNRPD3***	Small Nuclear Ribonucleoprotein D3 Polypeptide	This gene encodes a core component of the spliceosome, which is a nuclear ribonucleoprotein complex that functions in pre-mRNA splicing	1.2	0.027	0.7	8.30 × 10^−3^

**Table 2:** Shortlisted DEPs with characteristics of promising biomarkers enriched in the non-responder group compared to the responder group. Genes with a ROC *p*-value less than 0.05, an AUC greater than 0.7, and a Mann–Whitney *p*-value less than 0.05 were characterized as promising biomarkers.

Genes	Gene Name	Biological Function	FOLD Change	Mann–Whitney Test *p*-Value	AUC	ROC *p*-Value
***COPB1***	COPI Coat Complex Subunit Beta 1	This gene encodes a protein subunit of the coatomer complex to mediate biosynthetic protein transport from the ER via the Golgi up to the trans-Golgi network	1.1	0.0061	0.749	4.00 × 10^−4^
***MGLL***	Monoglyceride Lipase	Catalyzes the conversion of monoacylglycerides to free fatty acids and glycerol, and gene expression may play a role in cancer tumorigenesis and metastasis	1.3	0.0083	0.74	1.10 × 10^−3^
***HAS1***	Hyaluronan Synthase 1	Essential to hyaluronan synthesis, it has a structural role in tissue architectures and regulates cell adhesion, migration, and differentiation.	1.9	0.012	0.729	1.80 × 10^−3^
***TALDO1***	Transaldolase 1	Transaldolase 1 is a key enzyme of the nonoxidative pentose phosphate pathway, providing ribose-5-phosphate for nucleic acid synthesis and NADPH for lipid biosynthesis.	1.2	0.012	0.729	2.30 × 10^−3^
***DNAH9***	Dynein Axonemal Heavy Chain 9	This gene encodes the heavy chain subunit of axonemal dynein, a large multi-subunit molecular motor.	1.4	0.013	0.724	2.90 × 10^−3^
***KDELR3***	KDEL Endoplasmic Reticulum Protein Retention Receptor 3	This gene encodes a member of the KDEL endoplasmic reticulum protein retention receptor family	1.6	0.015	0.722	4.10 × 10^−3^
***HLA-DPB1***	Major Histocompatibility Complex, Class II, DP Beta 1	It plays a central role in the immune system by presenting peptides derived from extracellular proteins	1.8	0.02	0.709	5.20 × 10^−3^
***RBP3***	Retinol-binding Protein 3	Interphotoreceptor retinol-binding protein is found primarily in the interphotoreceptor matrix of the retina between the retinal pigment epithelium and the photoreceptor cells *	1.8	0.022	0.708	5.30 × 10^−3^
***STAP2***	Signal-transducing Adaptor Family Member 2	This gene encodes the substrate of breast tumor kinase, an Src-type non-receptor tyrosine kinase	1.2	0.025	0.703	6.90 × 10^−3^

* According to the Human Protein Atlas, several cases of colorectal cancers displayed moderate cytoplasmic and membranous staining [21].

**Table 3:** Drug targets among top proteins that are differentially overexpressed in the group of non-responders.

Gene	Protein Name Encoded by Gene	Drug	DRUGBANK ID	Drug Type	Usage	Drug Approval Status	Welch *t*-Test *p* Value
***QPRT***	Quinolinate Phosphoribosyltransferase	Niacin	DB00627	B vitamin	Hyperlipidemia, dyslipidemia, hypertriglyceridemia	Approved, investigational, nutraceutical	4.43 × 10^−5^
***CLCA4***	Chloride Channel Accessory 4	Talniflumate	DB09295	Small molecule, CaCC blocker, and Cl^−-^/HCO_3_^−^exchange inhibitor	Cystic fibrosis, chronic obstructive pulmonary disease (COPD), and asthma	Experimental	1.11 × 10^−2^
***ATG4B***	Autophagy-related 4B Cysteine Peptidase	Esomeprazole	DB00736	Proton pump inhibitor	Gastroesophageal reflux disease (GERD) for gastric protection to prevent recurrence of stomach ulcers or gastric damage	Approved, investigational	1.89 × 10^−4^
***ATG4B***		Nimodipine	DB00393	Calcium channel blocker	Acts primarily on vascular smooth muscle cells; improves the neurologic outcome following subarachnoid hemorrhage from ruptured intracranial aneurysm.	Approved, investigational	
***PTGS2***	Prostaglandin Endoperoxide Synthase 2	Diclofenac, Acetylsalicylic acid, Ibuprofen, Rofecoxib, Acetaminophen	DB00586, DB00945, DB01050, DB00533, DB00316	COX inhibitors, anti-inflammatory agents	Therapy for acute and chronic pain and inflammation from a variety of causes	Approved	2.08 × 10^−4^

**Table 4:** Clinical data of the study cohort.

Characteristics		Responders N (%)	Non-Responders N (%)	*p*-Value *
**Gender**	**Male**	3 (33.3)	7 (63.6) 4 (36.4)	0.4
	**Female**	6 (66.7)		
**Age (years)**	**Mean (SD)**	64.0 (6.7)	62.4 (10.3)	1.0
	**Median (Range)**	66.0 (50–72)	64.0 (48–83)	
**UICC staging**	**II**	0.0 (0)	2.0 (18.2)	0.5
	**III**	9.0 (100)	9.0 (81.8)	
**Tumor grade**	**1**	7.0 (77.8)	8.0 (72.7)	0.8
	**2**	2.0 (22.2)	3.0 (27.3)	
	**3**	0.0 (0.0)	0.0 (0.0)	
**Tumor localization**	**Inferior rectum (<5 cm)**	5.0 (55.6)	9.0 (81.8)	0.4
	**Mid rectum (5–10 cm)**	4.0 (44.4)	2.0 (18.2)	
	**Superior rectum (>10 cm)**	0.0 (0)	0.0 (0)	
**Acute toxicity**	**without**	1.0 (12.5)	2.0 (18.2)	0.7
	**with**	8.0 (87.5)	9.0 (81.8)	
**Tumor Regression Grade (TRG)**	**1**	8.0 (88.89)	/	
	**2**	1.0 (11.11)	/	
	**3**	/	2.0 (18.18)	
	**4**	/	7.0 (63.64)	
	**5**	/	2.0 (18.18)	
**Total**		9 (100)	11 (100)	

* Chi-squared test with Yates correction (two-tailed).

## Data Availability

The mass spectrometry proteomics data have been deposited to the ProteomeXchange Consortium via the PRIDE partner repository (https://www.ebi.ac.uk/pride/) with the dataset identifier PXD040451.

## Supplementary Materials

The following supporting information can be downloaded at https://www.mdpi.com/article/10.3390/ijms242015412/s1.
