# Supplementary material for: Data-Independent Acquisition Mass Spectrometry Analysis of FFPE Rectal Cancer Samples Offers In-Depth Proteomics Characterization of the Response to Neoadjuvant Chemoradiotherapy
Source: Int J Mol Sci. 2023 Oct 21;24(20):15412. doi: 10.3390/ijms242015412 (PMC10607861; doi:10.3390/ijms242015412)
Supplement: Supplementary file 1 [file ijms-24-15412-s001.zip › Supplementary material.pdf]

Supplementary material

File S1. Sample ID with corresponding category (responder/non-responder) and SDS page after protein separation

Table S1. Identification and quantification of proteins with statistics.

Table S2. Identification and quantification of 915 DEPs with statistical significance ($p<0.05$; S0=0.1) between responders and non-responders.

Table S3. Identification and quantification of 384 DEPs with more stringent statistical significance ($p<0.01$; S0=0.1) between responders and non-responders.

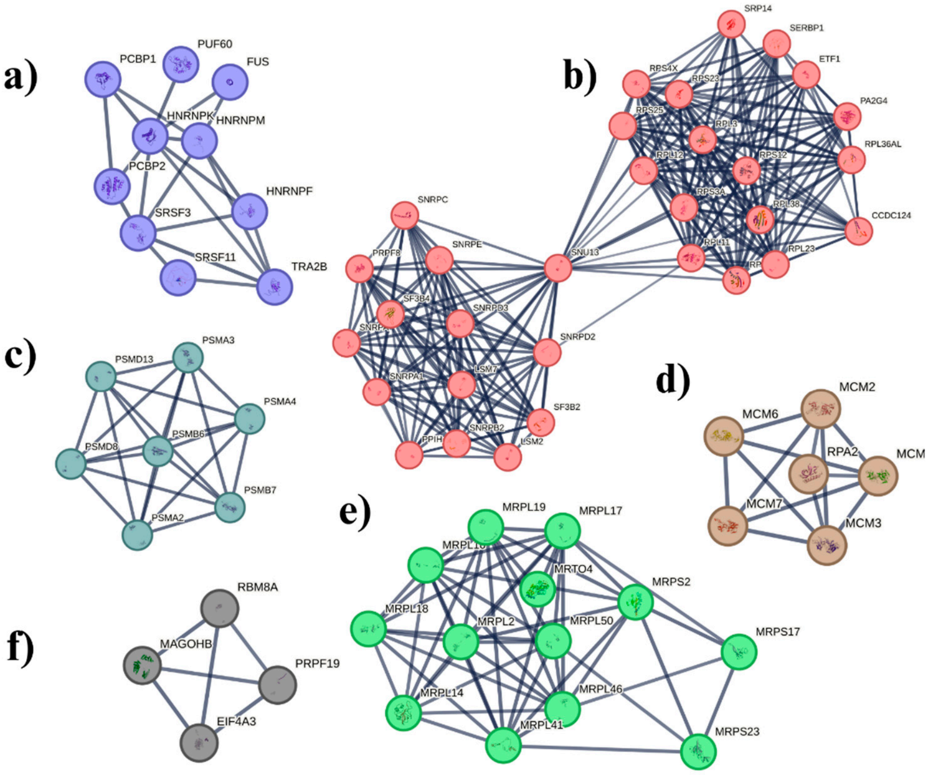

Figure S1. The protein-protein interaction (PPI) network analysis of differentially expressed proteins in responder group using STRING database. The 215 differentially expressed proteins were input into STRING database for PPI network analysis, and achieved a PPI network of 215 nodes and 797 edges, with PPI enrichment p-value < 1.0E10-16. The network was analyzed by app MCODE in Cytoscape and six clusters were detected, and the nodes in each cluster were input into STRING database to obtain functional and physical protein associations with highest confidence 0.9.

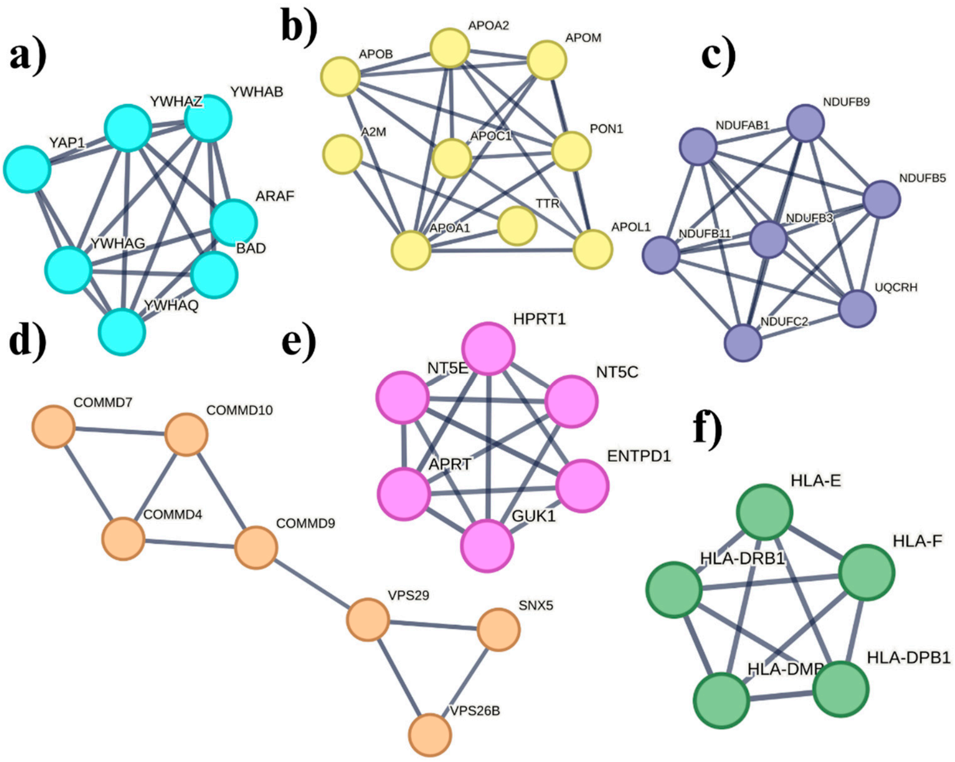

Figure S2. The protein-protein interaction (PPI) network analysis of differentially expressed proteins in nonresponder group using STRING database. The 700 differentially expressed proteins were input into STRING database for PPI network analysis, and achieved a PPI network of 700 nodes and 394 edges, with PPI enrichment p-value < 1.0E10-16. The network was analyzed by plug-in MCODE in Cytoscape and six clusters were detected, and the nodes in each cluster were input into STRING database to obtain functional and physical protein associations with highest confidence 0.9.
